# A novel method of modifying immune responses by vaccination with lipiodol-siRNA mixtures

**DOI:** 10.1186/1479-5876-4-2

**Published:** 2006-01-03

**Authors:** Thomas E Ichim, Igor A Popov, Neil H Riordan, Hamid Izadi, Zaohui Zhong, Li Yijian, Salman Sher, Eugenia K Oleinik

**Affiliations:** 1 Medistem Laboratories Inc, Tempe Arizona, USA; 2 Department of Surgery, University of Western Ontario, London, Ontario, Canada; 3 The Second Xiangya Hospital of Central South University, Changsha, China; 4 Division of Cardiology, Emory University, Atlanta, USA; 5 Institute of Biology, Karelian Research Center, Russian Academy of Sciences, Petrozavodsk, Russia

## Abstract

The dendritic cell (DC) possesses the ability to stimulate both T helper 1 (Th1) and Th2 responses depending on activation stimuli. Although it is known that chemically or genetically modified DC can be used therapeutically to steer immune responses towards either Th1 or Th2, cellular therapy with ex vivo manipulated DC is clinically difficult. Here we demonstrate a novel method of switching immune responses from Th1 to Th2 through in vivo immune modulation by administration of siRNA. We demonstrate that siRNA targeting of the IL-12p35 gene leads to a Th2 bias in vitro through an IL-10 dependent mechanism. In vivo administration of siRNA admixed with the oil-based contrast agent lipiodol in the presence of antigen and adjuvant induced a deviation in recall response to reduced production of IFN-γ and augmented IL-4 response using either KLH or ovalbumin. This simple method of in vivo modification of immune response possesses therapeutic potential in Th1-mediated diseases such as multiple sclerosis and autoimmune diabetes.

## Introduction

It is known that the immune response can be polarized into two broad subsets based on cytokine secretion of the T helper cells. T helper-1 cells (Th1) responses are associated with anti-viral and anti-cancer immune reactions and are characterized by high secretion of the cytokine IFN-γ and IL-2 [1]. In contrast, Th2 responses are effective for clearing parasitic infections such as schistosoma mansoni and are identified by high secretion of IL-4 and IL-13 [2]. In various human diseases a pathological polarization of the immune responses either to Th1 or Th2 is known to occur. For example, autoimmune diseases such as diabetes [3], multiple sclerosis [4], or arthritis [5], are associated with overproduction of Th1 cytokines. The causative role of these cytokines is noted by amelioration of animal models of autoimmune disease through treatment with inhibitors of such cytokines, such as antibodies or soluble receptors; however, clinical utilization is often impeded by systemic, non-desired side effects. Therefore a desired method of treating autoimmune diseases associated with Th1-hyperactivities would be targeting the actual initiating event that triggers such immune reactions.

It is known that naïve CD4^+ ^T helper cells (Th0) are programmed by the antigen presenting cell in order to become polarized into either the Th1 or Th2 phenotype [6]. More recent studies have demonstrated that dendritic cells (DC) play a key role in recognizing the exogenous pathogens, and making the decision of whether a Th1 or Th2 response is needed [7]. For example, parasites contain distinct chemical signatures that are recognized by specialized toll-like receptors (TLR) on DC, which instruct the DC to promote the appropriate response. In the case of schistosomiasis, the schistosomal egg antigen (SEA) is known to bind to TLR-2 on DC, and stimulate production of IL-13, which in turn shifts the Th0 cell into a Th2 cells [8,9]. Conversely, Gram-negative bacteria contain lipopolysaccaride (LPS), which activates TLR-4, leading to production of IL-12 by the DC, which then stimulates Th1 differentiation [10]. Since it is established that Th1 immunity is essential for clearance of many Gram-negative bacteria, it appears that the DC plays a "central cognitive" role in guiding the immune response. Conversely, manipulation of this cell type would allow us to selectively modulate immune responses to either Th1 or Th2. Since DC can be pulsed with antigen, the manipulation of antigen-pulsed DC would allow for selective alterations of immunity in an antigen-specific manner. Although this has been performed in animal models of autoimmunity [11-15], the clinical utilization is hampered by the need for ex vivo cellular manipulation, which is expensive and difficult.

RNA interference (RNAi) is the cellular defense mechanism by which a double-stranded RNA specifically silences mRNA transcripts with strict homology to the double strand [16]. The silencing effect of exogenous double-stranded RNA is very potent in that it can mediate gene-silencing even at a concentration of 1–3 double strands per cell [17]. In light of this potency and selectivity, the induction of RNAi in DC would allow for silencing of genes that stimulate either Th1 or Th2. Since it is known that inhibition of Th1 cytokines in DC results in the stimulation of Th2 immunity and vice versa, we hypothesize that silencing of IL-12 would result in DC with a propensity of stimulating Th2 immune responses.

While we and others have been able to modify immunological parameters of DC through silencing IL-10 [18], IL-12p35 [19], IL-12p40 [20], T-bet [21] and SOCS1 [22], a method of easily inducing immune modulation in vivo is lacking. In this study we induce an "in vivo genetic modification", presumably through DC using an immobilizing agent (CFA), a transfection associated agent (lipidiol) and antigen. We demonstrate inhibition of Th1 and upregulation of Th2 responses using this simple immunization protocol.

## Materials and methods

### Animals

Female C57/BL6 and BALB/c mice (The Jackson Laboratories, Bar Harbor, ME), 5 wk of age, were kept in filter-top cages at the Animal Care and Veterinary Services Facility, the University of Western Ontario according to the Canadian Council for Animal Care Guidelines. Mice were fed by food and water ad libitum and allowed to settle for 2 wk before initiation of experimentations.

### DC generation and siRNA transfection

At Day 0, bone marrow cells were flushed from the femurs and tibias of C57/BL6 mice, washed and cultured in 6-well plates (Corning, NY) at 4 × 10^6 ^cells/well in 4 ml of complete medium (RPMI 1640 supplemented with 2 mM L-glutamine, 100 U/ml penicillin, 100 μg of streptomycin, 50 μM 2-ME, and 10% FCS (all from Life Technologies, Ontario, Canada) supplemented with recombinant GM-CSF (10 ng/ml; PeproTech, Rocky Hill, NJ) and recombinant mouse IL-4 (10 ng/ml; PeproTech). All cultures were incubated at 37°C in 5% humidified CO_2_. Non-adherent cells were removed after 48 h of culture (Day 2) and fresh medium was added. After 7 days of culture, >90% of the cells expressed the characteristic DC-specific marker CD11c as determined by FACS. DC were washed and plated in 24-well plates at a concentration of 2 × 10^5 ^cells/well in 400 μl of serum-free RPMI 1640. Transfection with GenePorter, lipiodol, or naked siRNA was performed as described below on day 7 of culture.

### siRNA synthesis and transfection

siRNA sequences were selected according to the method previously used by us [19]. siRNA specific for IL-12p35 (AACCUGCUGAAGACCACAGAU) or mismatched (mixed) control sequence (AACTGCCAGATGGATGGTGAC) were synthesized and annealed by the manufacturer (Dharmacon, Lafayette, CO) and added at a concentration of 60pMol to DC cultures. For transfection, 3 μl of 20 μM annealed siRNA were incubated with 3 μl of GenePorter (Gene Therapy Systems, San Diego, CA) or lipiodol (Ultra-Fluide™ Laboratoire Guerbet, France) in a volume of 100 μl of RPMI 1640 (serum free) at room temperature for 30 min. This was then added to 400 μl of DC cell culture as described above. Mock controls were transfected with 3 μl of GenePorter alone. For naked siRNA, addition of the same concentration of siRNA was performed and procedures were repeated in an identical manner with the exception of addition of transfection reagent. After 4 h of incubation, an equal volume of RPMI 1640 supplemented with 20% FCS was added to the cells. Twenty-four hours later, transfected DC were washed and used for subsequent experiments. DC activation was performed in 24-well plates by stimulation with LPS (10 ng/ml; Sigma-Aldrich, St. Louis, MO) plus TNF- (10 ng/ml; PeproTech) for 24 hours.

### Flow cytometry

Phenotypic analysis of DC was performed using flow cytometry on a FACScan (Becton Dickninson, San Jose, CA) and analyzed using CellQuest software (BD Biosciences). The cells were stained with FITC-conjugated mAb against surface markers associated with DC maturation: anti-mouse CD11c, anti-mouse CD40, anti-mouse CD80, and anti-mouse CD86 (Cedarlane Laboratories, Mississauga, ON). Ig of the same isotype were used as controls. Annexin V and propidium iodine analysis for apoptosis, necrosis was performed using the Apotag kit (Cedarlane Laboratories, Hornby Ontario, Canada).

### Mixed lymphocyte reaction

C57/BL6 DC after transfection were irradiated (3,000 rad) and seeded in triplicate at various concentrations in a flat-bottom 96-well plate (Corning) for use as stimulator cells. Splenic T cells from BALB/c mice were isolated by gradient centrifugation over Ficoll-Paque (Amersham Pharmacia Biotech, Quebec) and T cell nylon wool column purification, and added as responders (5 × 10^5 ^cells/well). The mixed lymphocytes were cultured at 37°C for 72 h in 200 μl of RPMI 1640 supplemented with 10% FCS, 100 U/ml of penicillin, and 100 μg/ml of streptomycin and pulsed with 1 μCi/well of ^3^H-labelled thymidine (Amersham Pharmacia Biotech) for the last 16 h of culture. Cells were harvested onto glass fiber filters, and the radioactivity incorporated was quatitated using a Wallac Betaplate liquid scintillation counter (Beckman, Fullerton, CA). Results were expressed as the mean counts per min of triplicate cultures ± SEM. In some experiments anti-IL-10 (JES5 2A5, Pharmingen) or isotype control antibody were added for the duration of the MLR at a concentration of 5 ug/ml.

### Immunization with Ag/Lipiodol mixture

C57/BL6 mice were immunized intradermally at the interior side of both hind legs with 100 μl of KLH or ovalbumin (1 μg/μl) emulsified in CFA (Difco Laboratories, Detroit, MI) in the presence or absence of 10 nMol siRNA and 10% lipiodol. After 14 days mice were sacrificed and T cells extracted as described above.

### Proliferation assays

Proliferative recall responses to KLH and ovalbumin in immunized mice were assessed by sacrificing C57/BL6 mice 14 days after immunization with antigen-loaded DC. T cells were purified from suspensions of lymph nodes using CD4+ T cell column (R&D Systems) after washing in PBS. Purified T cells were cultured in 96 well plates with irradiated syngeneic splenocytes in triplicate and mixed with serial dilutions of KLH or OVA at concentrations ranging from 0–10 ug/ml. Following a 72-h incubation, 1 μCi of [^3^H]thymidine (Amersham) was added to each well for 16 h. Using an automated cell harvester, the cells were collected onto glass microfiber filter, and the radioactive labeling incorporation was measured by a Wallac Betaplate liquid scintillation counter.

### ELISA

The supernatants from recall response T cell cultures or MLR were harvested and assessed for DC cytokines (IL-12p70, IL-10) and T cell cytokines (IFN-, IL-4) by ELISA. Cytokine-specific ELISA (Endogen, Rockford, IL) was used for detecting cytokine concentrations in culture supernatants according to the manufacturer's instructions using a Benchmark Microplate Reader (Bio-Rad, Hercules, CA).

## Results

### Lipiodol enhances transfection of functional siRNA into DC

We have previously demonstrated that transfection of DC with siRNA specific for the p35 component of IL-12 induces potent gene specific silencing at the mRNA transcript level as demonstrated by RT-PCR and subsequently reduced expression of the IL-12 p70 heterodimer as witnessed by ELISA protein [19]. Furthermore, we and others have reported that siRNA can be endocytosed into dendritic cells (DC) and other cell types in absence of transfection reagent both in vitro and in vivo [23-25]. Since lipiodol is a clinically used contrast agent with ability to mediate transfection of siRNA[26] in vivo, we chose to investigate whether lipiodol can be used to increase uptake of siRNA in DC in using an in vitro system.

**Figure 1:**
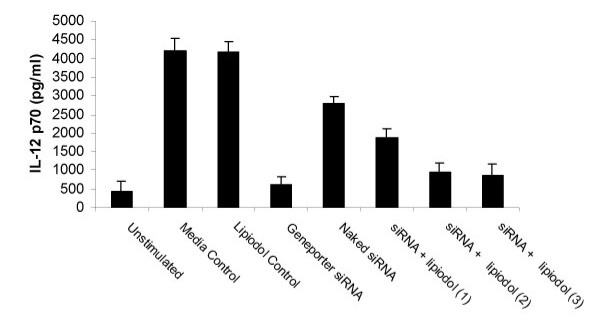
Lipiodol can serve as a transfection reagent. Day 7 bone marrow derived DC were cultured alone, or with IL-12 p35 specific siRNA delivered by optimized GenePorter concentration (3 μl/culture) or 3 concentrations of lipiodol (1,2, or 3 μl/culture). Following a 24 hour incubation cells were plated at 1 × 10^6 ^in 6 well culture dishes and activated for 24 hours with 10 ng/ml LPS and 10 ng/ml TNF-*α*. Supernatant was harvested and analyzed by ELISA for IL-12 p70 production.

In our laboratory, the combination of 10 ng/ml of LPS and TNF-α, respectively, (LPS/TNF) is used as a standard method of inducing activation of bone marrow derived DC for production of IL-12, as well as upregulation of costimulatory molecules such as CD40, CD80 and CD86 [27]. Using this stimulation system, and assessing IL-12 p70 production by ELISA, we sought to determine the potency of mixed lipiodol with siRNA to p35 at suppressing IL-12 production. We administered naked siRNA, and siRNA in various concentrations of lipiodol to day-7 bone marrow derived C57/BL6 DC. Activation by LPS/TNF was performed on day 8 while culture supernatants were assessed for IL-12 production on day 10 of culture. We observed that siRNA transfection with GenePorter induced a potent (>90%) inhibition of IL-12 production and that the naked siRNA induced a smaller (>30%) inhibition. Transfecting the siRNA with lipiodiol at concentration of 2 and 3 μl/well induced a significantly stronger inhibition of IL-12 production (>75%) as compared to naked siRNA, but not to the same extent as GenePorter (Figure 1). Similarly to our previously published experiments, administration of mismatched siRNA had no inhibitory effect on production of IL-12 (data not shown). Furthermore, although it has previously been reported that lipiodol is not cytotoxic even at high concentrations [28], we wanted to discount the possibility that lipiodol was mediated non-specific killing of DC. Viability assays using annexin-V and PI staining and analyzed by flow cytometry demonstrated no increase in apoptosis or necrosis in comparison to untreated DC (data not shown). Overall, these data support the notion that lipiodol is an easy to use method of transfecting bone marrow derived DC in vitro DC.

#### Immune modulation by lipiodol/siRNA

**Figure 2:**
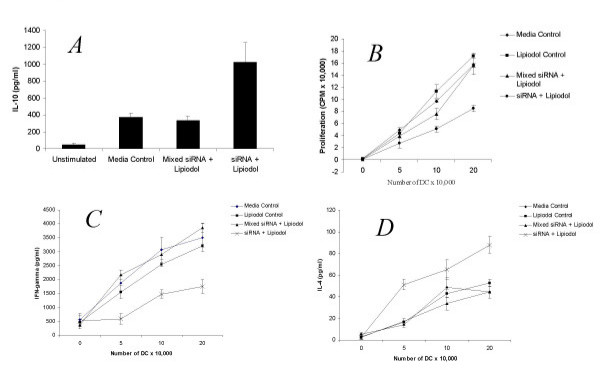
Lipiodol transfected DC are immune modulatory. *A*. Day 7 bone marrow derived DC were cultured alone or transfected with mismatched siRNA, or IL-12p35-specific siRNA using 3 μl/culture lipiodol. Following a 24 hour incubation cells were plated at 1 × 10^6 ^in 6 well culture dishes and activated for 24 hours with 10 ng/ml LPS and 10 ng/ml TNF-*α*. Supernatant was harvested and analyzed by ELISA for IL-10 production. *B*. C57/BL6 DC were transfected with mismatched siRNA, IL-12p35-specific siRNA or lipiodol alone, irradiated (3,000 rad) and seeded in triplicate at various concentrations in a flat-bottom 96-well plate. Splenic T cells from BALB/c mice were added as responders (5 × 10^5 ^cells/well). The mixed lymphocytes were cultured for 72 h and proliferation was assessed by thymidine incorporation. *C *&*D*. IFN-γ and IL-4 concentrations, respectively, were assessed from MLR cultures at 48 hours of incubation.

We have previously demonstrated that DC silenced for the IL-12p35 subunit possess an increased production of IL-10 and are poor stimulators of MLR [19]. Using the 3 ul concentration of lipiodol that we found most effective at inhibiting IL-12 production in Fig 1, we verified whether the lipiodol/siRNA treated DC possessed the same immunomodulatory properties as previously reported by us. Indeed, we observed that siRNA transfected by lipiodol induced a specific increase in IL-10 production by LPS/TNF stimulated DC as seen in Fig 2a. Since the specific function of DC in vivo is stimulation of T cell responses, we sought to determine whether the siRNA/lipiodol mixture had effects on inhibition of mixed lymphocyte reaction (MLR). Indeed, the siRNA specific for IL-12 p35 and not the mismatched (mixed) control inhibited proliferation of responding T cells in a 3 day MLR with naïve BALB/c splenocytes. Furthermore, as seen in Fig 2b the lipiodol alone did not suppress allostimulatory ability of the DC, indicating that the siRNA itself was specifically inducing inhibition. When supernatants of the MLR were assayed for the prototypic Th1 and Th2 cytokines IFN-γ and IL-4, respectively, an inhibition of IFN-γ Fig 2c, and upregulation of IL-4 Fig 2d was observed. This data is in agreement with our previous work in which we demonstrated Th1 to Th2 immune modulation by silencing of the IL-12 p35 subunit [19]. Furthermore, the current data supports the use of lipiodol as a transfection reagent for induction of immune modulation by siRNA.

#### Th1 > Th2 shift is associated with IL-10 production

**Figure 3:**
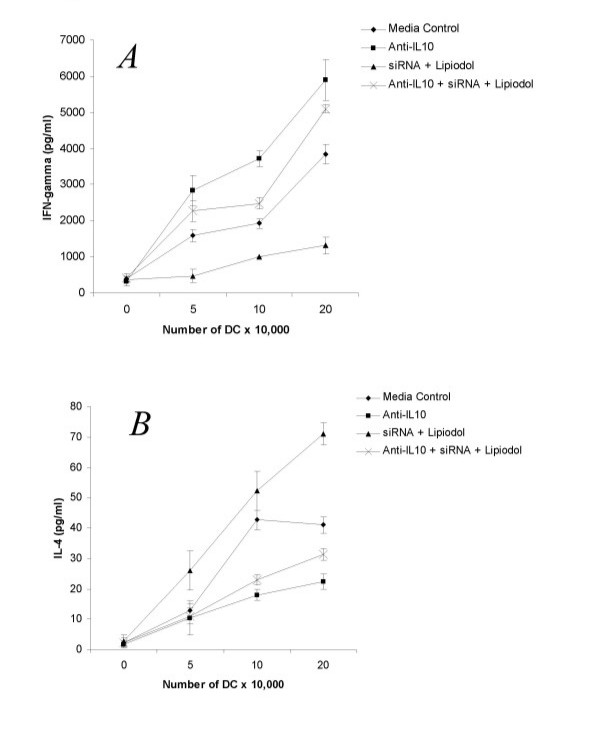
IL-10 is mediates immune modulation by IL-12 silenced DC. *A*. MLR was performed with various concentrations of irradiated C57/BL6 stimulator DC transfected with siRNA to IL-12p35 or control transfected DC, and BALB/c responder T cells. 5 μl/ml of anti-IL-10 (JES5 2A5) antibody was added throughout the culture time. Supernatant was collected from 48-hour MLR cultures and assessed for IFN-γ or IL-4 (*B*) by ELISA.

In our previous studies we have observed that silencing IL-12 is associated with an upregulation in IL-10 production [19]. While IL-10 has previously been described to be a Th2 cytokine [29], it has recently been implicated in generation of CD4^+ ^CD25^+ ^T regulatory cells [30] and TR1 cells [31]. We wanted to assess whether siRNA silencing mediated immune modulation is associated with autocrine IL-10 production by the DC. Indeed, it is known that immature and tolerogenic DC produce this cytokine in an autocrine fashion [32] and that gene-silencing of IL-10 on DC stimulates Th1 immunity and DC maturation [18]. With this in mind we added blocking anti-IL-10 antibodies to siRNA treated DC during stimulation of allogeneic BALB/c T cells in MLR. A dose-dependent inhibition in ability to stimulate Th1 responses was seen with addition of anti-IL-10. Specifically, the IL-12 silenced DC stimulated the responding T cells to decrease production of IFN-γ and increase production of IL-4 as depicted in Fig 3a and 3b respectively. Noteworthy is the observation that complete inhibition of Th1 stimulation was not achieved by anti-IL-10, implying that stimulation of IFN-γ and inhibition of IL-4 was not completely dependent stimulation of IL-10 and was due to other factors such as suppression of IL-12. Addition of isotype control antibody did not influence cytokine production or proliferation (data not shown).

While we previously demonstrated that IL-12 silenced DC possessed similar levels of costimulatory molecules as wild-type DC [19], we did not assess whether differences were present after stimulation of DC during the MLR process. This would be anticipated since activated T cells produce a variety of membrane bound (CD40L, CD134L), and soluble factors (IFN-γ, TRANCE) that stimulate DC maturation [33-37]. Extracting DC using magnetic activated cell sorting (MACS) after the MLR demonstrated significant increases in the mean fluorescent intensity of CD40, CD80 and CD86, however no difference was observed between IL-12 silenced or non-silenced DC. As seen in Table 1, it does not appear that IL-12 silencing leads to Th1>Th2 due to alternation in costimulatory molecule expression. This is particularly interesting since it is known that IL-10 suppresses CD40, CD80, and CD86 expression on DC [38]. Indeed, addition of anti-IL-10 antibody to the MLR significantly increased expression of all 3 costimulatory molecules Table 1. This is in agreement with higher expression of CD40, CD80, and CD86 in IL-10 knockout DC during the process of MLR stimulation as compared with wild-type DC [39].

**Table 1:** 

	**Surface molecules (MFI)**		
	CD80	CD86	CD40
**Unactivated DC**			
Media Control	571.7 (± 23)	1194 (± 125)	29.5 (± 1.5)
Lipiodol Control	567.3 (± 21)	1251 (± 173)	28.6 (± 1.3)
Mixed siRNA	572.5 (± 10)	1365 (± 157)	29.7 (± 1.3)
siRNA alone	559.6 (± 33)	1201 (± 193)	30.1 (± 0.9)
siRNA + Lipiodol	577.3 (± 15)	1195 (± 103)	30.4 (± 1.3)
**Post-MLR**			
Lipiodol Control	1962.9 (± 136)	2707.1 (± 576)	140.5 (± 35.4)
Mixed siRNA	1933.2 (± 324)	3031.6 (± 489)	170.3 (± 26.9)
siRNA alone	1905.7 (± 351)	2759.9 (± 392)	167.2 (± 46.9)
siRNA + Lipiodol	2050.8 (± 119)	3051.1 (± 335)	153.3 (± 36.7)
**Post-MLR + Anti-IL10**			
Lipiodol Control	3529 (± 302)	4862 (± 583)	236.0 (± 47.3)
Mixed siRNA	3442 (± 247)	4777 (± 773)	242.8 (± 58.9)
siRNA alone	3723 (± 453)	4863 (± 669)	237.4 (± 43.5)
siRNA + Lipiodol	3985 (± 339)	4957 (± 501)	254.3 (± 49.3)

Surface staining for FACS analysis was performed using anti-CD11c Ab (gated DC) and anti-CD80, CD86 or CD40 Abs (MFI values). Samples stained with the appropriate isotype-matched control Abs were analyzed in parallel to establish the gating criterion. Results are representative of at least three independent experiments for each culture condition.

#### Lipiodol/siRNA induces Th1>Th2 shift in vivo

**Figure 4:**
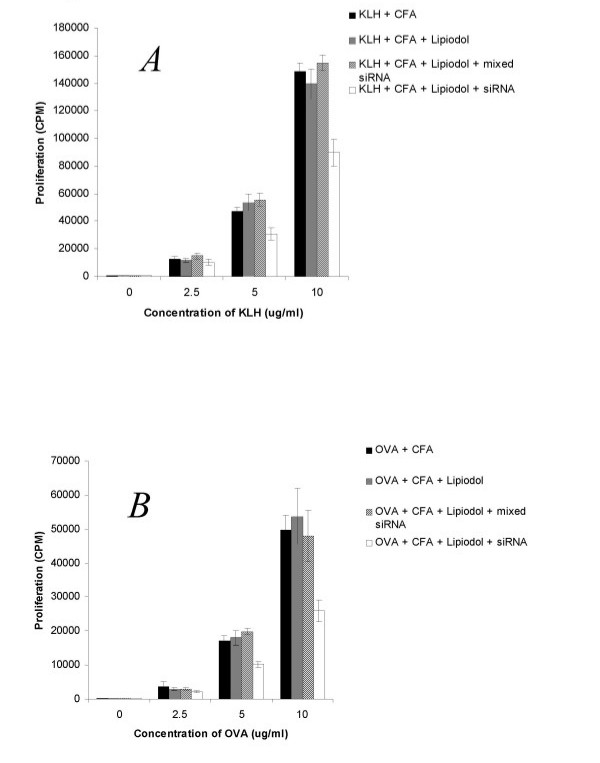
Inhibition of recall response by lipiodol/siRNA vaccine. *A*. C57/BL6 mice were immunized intradermally at the interior side of both hind legs with 100 μl of KLH or ovalbumin (1 μg/μl) emulsified in CFA in the presence or absence of 10 nMol siRNA and 10% lipiodol. After 14 days mice were sacrificed and T cell recall responses were assessed culturing purified CD4+ T cells with irradiated syngeneic splenocytes in triplicate and mixed with serial dilutions of KLH or OVA (*B*) at concentrations ranging from 0–10 ug/ml. Following a 72-h incubation, proliferation was assessed by thymidine incorporation.

**Figure 5:**
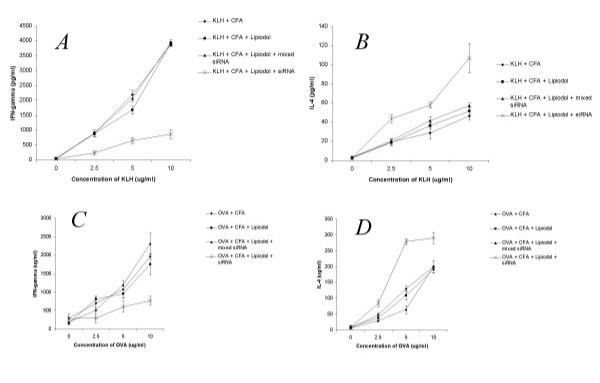
In vivo immune modulation lipiodol/siRNA vaccine. C57/BL6 mice were immunized intradermally at the interior side of both hind legs with 100 μl of KLH or ovalbumin (1 μg/μl) emulsified in CFA in the presence or absence of 10 nMol siRNA and 10% lipiodol. After 14 days mice were sacrificed and T cell cytokine responses were assessed culturing purified CD4+ T cells with irradiated syngeneic splenocytes in triplicate and mixed with serial dilutions of antigen. IFN-γ in KLH (A) and ovalbumin (C) cultures, and IL-4 in KLH (B) and ovalbumin (D) cultures was assessed by ELISA.

Having demonstrated above that lipiodol can serve as a transfection reagent for uptake of siRNA in DC, combined with the fact that lipiodol is commonly used for a variety of clinical applications [40,41], we assessed whether lipiodol/siRNA can be used to modulate immune responses in vivo. Accordingly, we used the KLH and OVA recall responses as an indicator. C57BL/6 mice were immunized with the IL-12 siRNA/lipiodol mixture combined with CFA and either KLH or OVA. 14 days following immunization, recall response experiments were performed using isolated CD4 T cells from draining lymph nodes as previously performed by us [19]. Proliferative responses for both antigens were significantly inhibited in the lipiodol/siRNA treated animals at restimulation concentrations of 5 and 10 ug/ml (Fig 4a and 4b). When supernatants were harvested and analyzed by ELISA for cytokine response, a Th1>Th2 shift was observed for both KLH and ovalbumin as demonstrated by higher production of IFN-γ (Fig 5a and 5c) and IL-4 (Fig 5b and 5d), respectively. In order to assess for antigen specificity, in some experiments, mice were immunized with siRNA/lipiodol with KLH and concurrently injected with mixed siRNA/lipiodol and OVA. The recall response to KLH was suppressed in terms of proliferation and possessed a Th2 cytokine profile, whereas the response to OVA was not immune modulated (data not shown). Other experiments not shown included the immunization in absence of lipiodol which did not result in any immunomodulatory effect of siRNA. Overall, these experiments strongly suggest that siRNA can be administered both in vitro and in vivo for immune modulatory purposes using lipiodol as a carrier.

## Discussion

Lipiodol (trade name Ultra-Fluide™) is an iodized poppy seed oil fatty acid ethylester commonly used as contrast media for radiography [42]. Commonly, lipiodol alone or with chemotherapy is administered to patients with hepatic cell carcinoma for embolization of tumor-feeding arteries, as well as localized delivery of chemotherapy [43]. The finding that lipiodol selectively is uptaken by endothelial cells through pinocytosis [28], combined with the fact that it has previously been used for siRNA delivery in vivo [26] stimulated us to question whether lipiodol can be used as a carrier for siRNA. We observed that although lipiodol was not as effective as GenePorter for in vitro transfection and gene silencing, the inhibitory effects where several fold more potent than the administration of naked siRNA.

We therefore continued to seek whether lipiodol transfer of siRNA can still mediate the immune modulatory effects previously reported by us in terms of switching Th1 to Th2 responses [24]. Although this was the case, we also observed that the switch was dependent on IL-10 production. This is somewhat interesting since although IL-10 is a Th2 cytokine, secretion of IL-10 has also been demonstrated to stimulate CD8 proliferation [44], and even to suppress atopy [45], a manifestation of classical Th2 responses. In this study our data supports a prototypic role of IL-10 secreted by DC in stimulation of IL-4 and IFN-γ production. We are however aware that assessment of other Th1/Th2 associated factors are needed to conclusively demonstrate a Th2 switch was occurring. Additionally, although in our hands, similar culture situations stimulate IL-10 production primarily by DC, we can not rule out the production of IL-10 by T cells. Future studies involve looking at T cell transcription factors such as T-bet and GATA-3, associated with Th1 and Th2 cells, respectively [46].

While we were able to induce in vivo immune modulation through administration of the lipiodol/siRNA mixture, we did not demonstrate that in vivo the DC were indeed the culprits for immune modulation. As previously stated, lipiodol in vivo is pinocytosed by endothelial cells [28], and most likely by other cells such as monocytes and macrophages. Indeed both endothelial cells, and macrophages have been demonstrated to possess immune modulatory activity. For example, antigen presentation by unmanipulated endothelium is known to mediate tolerance in various situations [47,48]. Indeed we conceived that the lipiodol itself may be inducing immune modulation due to specific targeting of antigen to endothelial presentation, however this was not the case since lipiodol together with antigen, or together with antigen and mixed siRNA did not mediate immune modulation. However, the fact that the endothelial cells themselves are protolerogenic may allow for synergy with the tolerogenic siRNA administered. The involvement of macrophages in the immune modulation witnessed is another possibility. Indeed, macrophages have been reported to acquire an M1/M2 phenotype similar to Th1/Th2 [49]. Preliminary experiments have indicated refractoriness to IL-12 production after LPS/TNF stimulation of CD11c DC derived from draining lymph nodes 2–3 days after administration of lipiodol/siRNA, however this will be the subject of an upcoming paper.

Regardless of the cellular mechanisms involved, the ability to immune modulate through administration of a "vaccine composition" with siRNA opens a whole world of novel possibilities both therapeutic and experiments. Through alleviating the need for ex vivo cellular processing, simple siRNA based vaccines can be developed for a variety of disease indications where a Th switch is desired. In terms of clinical applicability, the addition of siRNA for embolization of tumors would allow for induction of immune stimulation at the site of tissue necrosis. This would permit induction of immune responses to antigens released during the embolization procedure. Indeed similar approach used by Adema et al has demonstrated successful induction of immunity through tissue damage combined with immune stimulation [50].

In conclusion, we demonstrate that siRNA can be administered through a clinically useful method, thus opening the possibilities of genetic immune modulation without the use of viruses or ex vivo cellular manipulation.
